# Health Care Disparities Among Lesbian, Gay, Bisexual, and Transgender Youth: A Literature Review

**DOI:** 10.7759/cureus.1184

**Published:** 2017-04-20

**Authors:** Hudaisa Hafeez, Muhammad Zeshan, Muhammad A Tahir, Nusrat Jahan, Sadiq Naveed

**Affiliations:** 1 Clinical Observer, St. Michael's Hospital; 2 Department of Psychiatry, Bronx Lebanon Hospital Icahn School of Medicine at Mount Sinai, Bronx, NY; 3 Psychiatry, Suny Upstate Medical University, Syracuse, NY; 4 Psychiatry, Mount Sinai Chicago; 5 Psychiatry, KVC Prairie Ridge Hospital

**Keywords:** lgbt youth, healthcare disparities, lesbian, gay, bisexual, transgender

## Abstract

About 3.5% Americans identify themselves as lesbian, gay, or bisexual while 0.3% identify themselves as transgender. The LGBT (lesbian, gay, bisexual, and transgender) community belongs to almost every race, ethnicity, religion, age, and socioeconomic group. The LGBT youth are at a higher risk for substance use, sexually transmitted diseases (STDs), cancers, cardiovascular diseases, obesity, bullying, isolation, rejection, anxiety, depression, and suicide as compared to the general population. LGBT youth receive poor quality of care due to stigma, lack of healthcare providers’ awareness, and insensitivity to the unique needs of this community. The main objective of this literature review is to highlight the challenges faced by the LGBT youth and to enhance the awareness among physicians about the existing disparities in order to provide a more comprehensive, evidence-based, and humane medical care to this community.

## Introduction and background

Lesbian, gay, bisexual, and transgender (LGBT) is an umbrella term which includes a number of groups: lesbian (homosexual woman), gay (homosexual man or woman), bisexual (person who is attracted to both genders), transgender (person who identifies his gender as different from their biological one), queer (a synonym for gay; some people prefer to identify themselves as queer to empower themselves and take their identity “back from the bullies”), questioning (people who are unsure about their gender identity/sexuality), intersex (people with two sets of genitalia), asexual (people who are not sexually attracted to anyone and who don’t identify with any orientation), allies (the loving supporters of the community, though not necessarily part of it), two spirits (a tradition in many First Nations that considers sexual minorities to have both male and female spirits), and pansexual (person sexually attracted to others of any sex or gender).

It is noteworthy to mention that the lack of appropriate questions pertaining to gender and sexual identity in most national or state surveys makes it difficult to estimate the number of LGBT individuals and their health care needs. LGBT youth face a fear of coming out and discrimination because of their sexual orientation, gender identity, and gender expression [1]. It can lead to an increased risk of depression, posttraumatic stress disorder, substance use, and self-destructive behaviors. They are also particularly vulnerable to suicidal behaviors [1]. It is shocking to realize that LGBT youth represent up to 40% of all young people experiencing homelessness. They are also at an increased risk of physical or sexual abuse, STDs, and mental health issues [2-5]. We aim to highlight the factors leading to poorer health outcomes, social inequalities, and health care disparities in LGBT youth as compared to their heterosexual counterparts.

## Review

### Depression and suicidal risk among discriminated LGBT youth

Sexual minority adolescents report a greater incidence of mental health issues such as depression, anxiety, and increased suicidal behaviors than heterosexual adolescents [6]. In a school-based survey conducted in Boston, Massachusetts (n = 1320), 10% of participants identified themselves as LGBT and 58% of them were females. The respondents’ age ranged between 13 to 19 years in this survey. LGBT youth scored significantly higher on the scale for depressive symptomatology. They were also more likely than heterosexual and non-transgendered youth to report suicidal ideations (30% vs. 6%, p < 0.0001) and self-harm behaviors (21% vs. 6%, p < 0.0001) [7].

Data from the National Longitudinal Study of Adolescent Health revealed that sexual minority youth are prone to be isolated and disconnected from the social networks [8]. This estrangement can increase the risk of depressive symptoms among sexual minority males [8]. Similar trends were replicated in other community surveys [9]. Bisexual males and females were also more likely to express depressive symptomology than the heterosexual individuals [10].

### Sexual provocativeness and increased substance use

**Figure 1:**
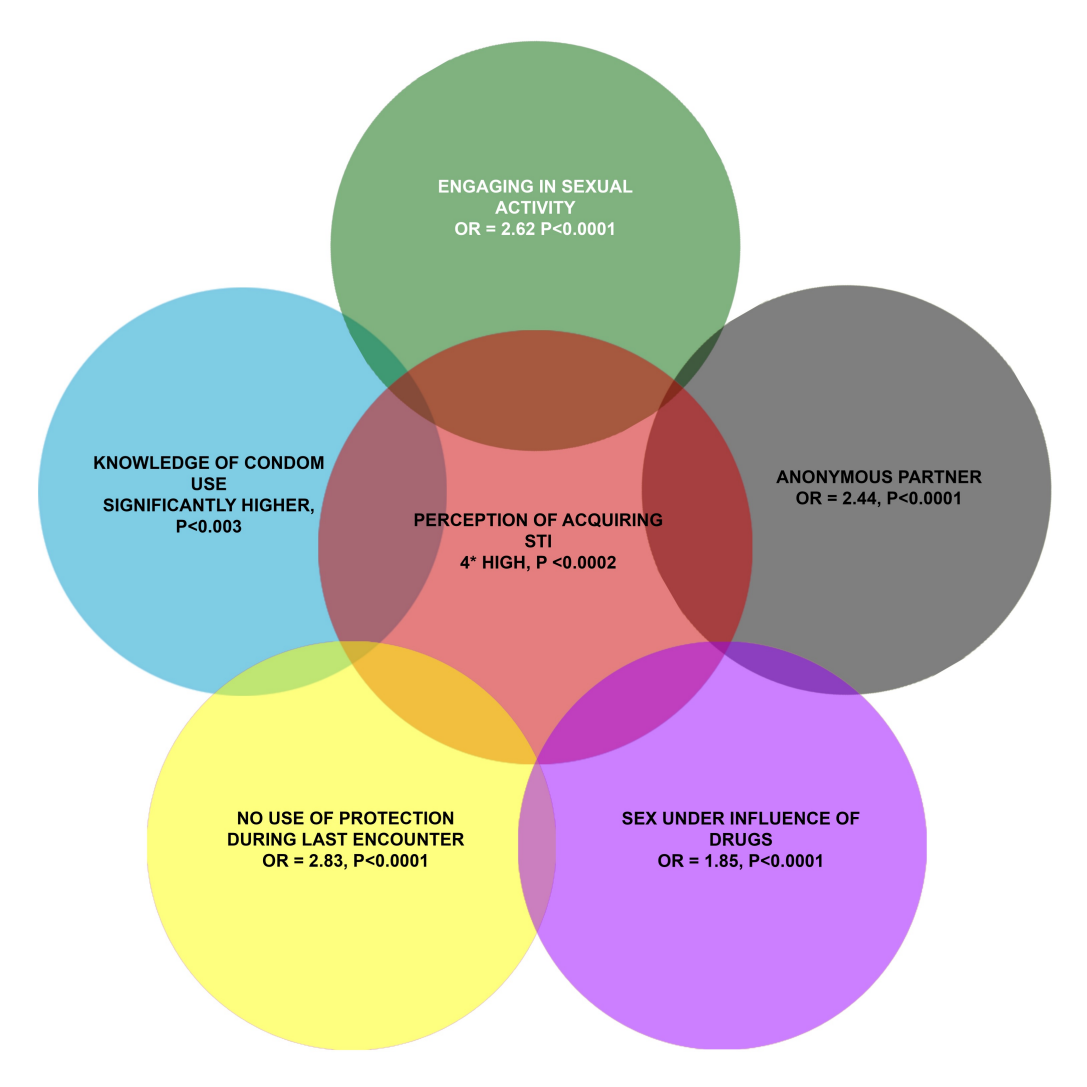
Odd ratios for LGBT youth versus heterosexual youth, Dane County Youth Assessment Surveys, Wisconsin, 2008-2009 LGBT: Lesbian, gay, bisexual, and transgender; STI: Sexually transmitted infection.

LGBT youth are more likely to engage in high-risk sexual behaviors leading to an increased incidence of STDs. The rates of gonorrhea, chlamydia, and HIV are twice as higher in sexually minority youth, as in heterosexual men [11]. According to the Dane County Youth Assessment Surveys (2008-2009), multiple factors accounted for unsafe sexual behaviors in LGBT youth including earlier age of sexual encounter, increased number of known and anonymous sexual partners, lack of education on safe sex practices, ineffective use of condoms, and testing and perception of STDs acquisition [12]. Figure 1 reflects the statistical values of these high-risk behaviors in LGBT youth.

A recent article in the American Journal of Public Health surveyed 13,174 individuals between 2005 and 2007. It reported similar concerning trends among sexual minority youth in terms of sexually provocative behaviors. They are at an increased risk for recreational substance abuse, obesity, and multiple cancer-related risk factors [13].

### Peer victimization and family rejection

Peer victimization is one of the leading causes of high-risk sexual behaviors in middle and high school students [12]. LGBT youth are frequently bullied at schools. They frequently get into fights, engage in truancy, and struggle with emotional distress and conduct problems. A longitudinal study showed that gay/bisexual boys are more likely to be victimized than heterosexual-identified boys (wave 1: odds ratio [OR] = 1.78, p = 0.011; wave 7: OR = 3.95, p = 0.001). Early victimization and emotional distress explained about 50% of disparities between LGBT and heterosexual youth in emotional distress in both boys and girls (each p < 0.015) [14].

The Youth Risk Behavior Survey (YRBS) in 2016 showed that 34% of LGBT teens were bullied in school, 18% stated having forced sex, 23% were the victim of sexual violence, and 18% struggled with physical violence [15]. Another survey replicated the above findings regarding peer victimization. This survey from 10 states and 10 large urban school districts in the United States revealed that sexual minority students were at a greater risk for being harassed, injury with a weapon, and bullying than heterosexual students [16]. The verbal and physical harassment along with other factors like concomitant substance use and family rejection can lead to an increased risk for suicidal ideations [17-20].

Another important issue is family rejection which was frequently observed in families with LGBT youth. The disclosure of their gender or sexual identity can cause significant interpersonal problems among youth and their families [21-22]. To further explore the impact of family acceptance or rejection, a study enrolled 245 LGBT Latino and non-Latino white young adults from the LGBT locale, whose sexual orientation was known to at least one parent. This study showed that family acceptance was more dependent on the family dynamics than the young person’s sexual orientation or gender identity. Parents belonging to Latino community, low socioeconomic status, religious affiliation, and immigrants were less accepting. In addition, family acceptance was associated with the positive self-esteem and good general health. It was protective against negative health outcomes such as depression, recreational substance use, and suicidal behaviors [23]. The Massachusetts High School survey also suggested that LGBT youth are either kicked out of their homes or feel uncomfortable about both staying and leaving. After becoming homeless, LGBT youth have an increased risk of poor physical mental health outcomes than heterosexual adolescents [24].

They also face enormous difficulty blending into the LGBT community once they recognize their gender and sexual identity. In one study, sexual minority women perceived smoking as one way of identifying with their peers [25].

### Increased risk of physical health problems, lack of gender/sex-specific health care, and office-based care for LGBT youth

The Center for Disease Control and Prevention data in 2014 showed that gay and bisexual men accounted for 83% of the new diagnoses of HIV among males, aged 13 years and older. The gay men were also at an increased risk of various cancers including prostate, testicular, anal, and colon, which might be related to limited culturally sensitive screening services [26-28]. There is also an increased risk of STDs like syphilis, human papillomavirus (HPV) infections, and hepatitis in MSM (Men who have Sex with Men). This literature also suggested an increased risk of breast, ovarian, and endometrial cancers in lesbians and bisexual women due to fewer full-term pregnancies, fewer mammograms, and obesity. The higher prevalence of obesity was found in lesbian women from the African American community and from low socioeconomic status [29].

Young LGBT individuals find it difficult to report their sexual identity to their clinicians. Some clinicians are not well trained in addressing the concerns of members of this community. A study conducted in Washington DC showed that 68% of sexual minority youth reported about not discussing their sexual orientation, and 90% reported reservations about reporting them to their clinicians [30]. Another study used purposive sampling to recruit nine women between the ages of 18 and 24 years who identified themselves as belonging to a sexual minority student group at a university in the Southwestern United States. Their audio interviews showed that the disclosure of sexual orientation and provider’s attitude were important influencing factors that negatively affected their experiences about health care delivery [31]. The lack of training can strain the therapeutic relationship between the providers and patients. Hence, it can influence the quality of care and appropriate delivery of health care.

### Summary

LGBT youth struggle with significant health care issues, in terms of increased disease prevalence as well as the lack of appropriate physicians’ training and health care disparities. The present literature review identifies evidence of poor well-being compared to their heterosexual counterparts. They experience frequent disadvantages from adolescence to adulthood. Reaching adolescence appears to be one of the hardest milestones. Resentment revolves around the subject of homophobia and heterosexism and it is not limited to their homes. They also struggle with these issues at schools and in the community. Peer victimization and isolation is highly prevalent in middle and high school settings [32-33]. Additionally, LBGT youth also face difficulties with acceptance into established LGBT communities. As a result, they tend to resort to unhealthy habits observed in some of these communities, and often face interpersonal violence [34]. LGBT youth is more likely to be involved in high-risk sexual behaviors at an earlier age than their heterosexual counterparts, and these behaviors are related to peer victimization, childhood physical and sexual abuse, substance use, and homelessness due to family rejection [35]. They are more prone to have an increased risk of depression, suicidal ideation, and substance use including tobacco, alcohol, cannabis, cocaine, ecstasy, methadone, and heroin [36].

Another important issue is discrimination in the delivery of health care at clinics and hospitals, where young LGBT individuals find it difficult to share their sexual identities with their clinicians. This lack of communication is responsible for the poor therapeutic alliance, lack of appropriate illness-related education, inadequate scheduled screening for communicable diseases, and inadequate interventions to prevent STDs [26].

All the stakeholders in the community need to develop a cohesive plan to deal with the challenges faced by this community. Parents, teachers, medical personnel, and the community as a whole can play a significant role in minimizing the disparities faced by LGBT youth. The policy makers can engage key stakeholders in formulating social and news media campaigns to address the social inequalities and lack of effective health care through the culturally appropriate messages. Parents and youth should be at the front and center of these interventions. Physicians should be culturally sensitive to meet the needs of LGBT youth. They should be trained adequately to provide nurturing, open communication, and empathic care to this population, in a respectful and nonjudgmental manner. There is also a need for more research to address the concerns of LGBT youth, including physical and mental well-being, social welfare, and employment opportunities.

## Conclusions

This literature review gives an insight on significant differences in the mental and physical health of the LGBT youth. Stigmatization, social stress, peer victimization, and family rejection are some of the concerning issues. The health care providers may lack adequate training on the specific needs and challenges faced by this community. This lack of training can perpetuate prejudice and discrimination, resulting in suboptimal medical care and an increased incidence of diseases and their risk factors. Healthcare providers need to be educated through proper training, and the guidelines need to be developed into practice, in order to provide a more comprehensive, scientific and humane care for this community.
